# Tolerance to a new free amino acid-based formula in children with IgE or non-IgE-mediated cow’s milk allergy: a randomized controlled clinical trial

**DOI:** 10.1186/1471-2431-13-24

**Published:** 2013-02-18

**Authors:** Roberto Berni Canani, Rita Nocerino, Ludovica Leone, Margherita Di Costanzo, Gianluca Terrin, Annalisa Passariello, Linda Cosenza, Riccardo Troncone

**Affiliations:** 1 Department of Paediatrics, European Laboratory for the Investigation of Food Induced Diseases (ELFID), University of Naples “Federico II”, Via S. Pansini, 5-80131, Naples, Italy; 2 Department of Women’s Health and Territorial Medicine, University “La Sapienza”, Rome, Italy; 3 Monaldi Hospital, Naples, Italy

**Keywords:** Food allergy, Faecal calprotectin, Eosinophilic cationic protein, Infant formula, Dietotherapy

## Abstract

**Background:**

Amino acid-based formulas (Aaf) are increasingly used in children with cow’s milk allergy (CMA). To be labeled hypoallergenic these formulas must demonstrate in clinical studies that they don’t provoke reactions in 90% of subjects with confirmed CMA with 95% confidence when given in prospective randomized, double-blind, placebo-controlled challenge (DBPCFC) trials. The majority of available safety data on Aaf derived from patients with IgE-mediated CMA. Considering substantial differences in the immunologic mechanism and clinical presentation of non-IgE-mediated CMA it’s important to investigate the hypoallergenicity of these formulas also in these patients. We prospectively assessed the tolerance to a new commercially available Aaf in children affected by IgE- or non-IgE-mediated CMA.

**Methods:**

Consecutive patients affected by IgE- or non-IgE-mediated CMA, aged ≤ 4 years, were enrolled. DBPCFC was carried out with increasing doses of the new Aaf (Sineall, Humana, Milan, Italy), using validated Aaf as placebo. Faecal concentrations of calprotectin (FC) and eosinophilic cationic protein (ECP) were monitored.

**Results:**

Sixty patients (44 male, 73.3%, median age 37, 95%CI 34.5–39.6 months, IgE-mediated CMA 29, 48.3%) were enrolled. At the diagnosis clinical symptoms were gastrointestinal (46.6%), cutaneous (36.6%), respiratory (23.3%), and systemic (10.0%). After DBPCFC with the new Aaf, no patient presented early or delayed clinical reactions. Faecal concentration of calprotectin and of ECP remained stable after the exposure to the new Aaf.

**Conclusions:**

The new Aaf is well tolerated in children with IgE- or non-IgE-mediated CMA, and it could be used as a safe dietotherapy regimen for children with this condition.

**Trial registration:**

The trial was registered in the ClinicalTrials.gov Protocol Registration System (ID number: NCT01622426).

## Background

Cow’s milk allergy (CMA) affects up to 3% of European children [1]. The management of children with confirmed CMA is based on complete avoidance of cow’s milk proteins (CMP) and leaves the physician with several dietary options, none of which, given the prevalence, spectrum and potential seriousness of the condition, can be recommended to all patients [2]. In the absence of an alternative to cow’s milk, the management of CMA is based on the use of safe, affordable and nutritionally adequate formulas. Extensively hydrolyzed cow’s milk protein formulas (eHF), which are considered as safe for most children with CMA, are still liable to contain residual peptides, and hypersensitivity reactions may occur in infants allergic to CMP [3]. Thus, the allergenicity of a specific product must be addressed on an individual basis before recommending a formula as a substitute for cow’s milk. Soy-based formula can also concomitant sensitize CMA children to soy [4-8]. Amino acid-based formulas (Aaf) have been studied from safety and nutritional efficacy perspectives [9-11]. These formulas have been proposed for subjects highly sensitive to CMP and that cannot be managed using eHF and for children with multiple food allergies [3,11]. In these conditions Aaf are able to effectively cure allergic symptoms and to improve body growth [12-14].

According to the American Academy of Pediatrics (AAP) Subcommittee on Nutrition and Allergic Diseases a hypoallergenic formula must be tested in infants and children with hypersensitivity to CMP, with findings verified by elimination-challenge tests under double-blind, placebo-controlled conditions (DBPCFC). These tests should show with at least 95% confidence that 90% of allergic individuals will not react to the formula [11]. To control for possible false-negatives, a negative DBPCFC should be followed by an open food challenge (OFC) with the tested formula [15].

The possible subclinical intestinal mucosa inflammation in children with CMA in response to a challenge with Aaf is largely unknown. Faecal calprotectin (FC) is a widely accepted non-invasive inflammatory marker able to measure even subclinical intestinal inflammation. The FC concentration correlates with the level of mucosal inflammation, and increased FC levels have been demonstrated in patients with food allergy [16-18]. Similarly, eosinophilic cationic protein (ECP) has been proposed for the diagnostic work up and follow-up of children with food allergy, even during the oral food challenge [19]. Starting from these considerations we decided to include the evaluation of these non-invasive markers to investigate possible subclinical intestinal inflammation induced by the study formula.

In this study we aimed to evaluate the tolerance to a new commercially available Aaf containing nucleotides in children with documented IgE- or non-IgE-mediated CMA based on the criteria developed by the AAP’s Subcommittee on Nutrition and Allergic Diseases [3].

## Methods

### Trial design

The study was conducted from October 2008 to June 2010. The research protocol and informed consent form were approved by the Ethics Committee of the University of Naples “Federico II”. We evaluated patients (aged ≤ 4 years) under good clinical control with a sure diagnosis of IgE- or non-IgE-mediated CMA, confirmed by the result of DBPCFC performed in the last 2 months. The vast majority of these patients received the diagnosis during the first year of life, but all subjects were still reacting to CMP. When the last oral food challenge was performed the vast majority (93.1%) of patients with IgE-mediated CMA reacted to the first 4 doses (from 0.1 to 3 ml of cow’s milk) and all patients with non-IgE-mediated CMA reacted within 48 hours after the procedure. We defined IgE- or non-IgE-mediated CMA subjects according to the clinical history (acute or delayed onset of symptoms after ingestion of CMP), the result of oral food challenge (occurrence of typical symptoms within 2 or more than 2 hours after the administration of the last dose), and the result of serum specific IgE, skin prick tests (SPTs) and atopy patch tests (APTs).

The subjects were referred to 2 tertiary paediatric gastroenterology and allergy Centers. We excluded children with concomitant chronic systemic diseases, congenital cardiac defects, active tuberculosis, autoimmune diseases, immunodeficiency, chronic inflammatory bowel diseases, celiac disease, cystic fibrosis, metabolic diseases, lactose intolerance, malignancy, chronic pulmonary diseases, malformations of the gastrointestinal tract, gastroesophageal reflux disease non-food allergy-related, suspected eosinophilic gastrointestinal disorders. At the first evaluation, the clinical history, information on food allergy and baseline clinical conditions were assessed by 3 experienced paediatric allergists in each Center and the child was invited to participate in the study. At least one parent of each subject provided written informed consent. Children with atopic dermatitis were included if it was under sufficient control to allow recognition of a positive response to the challenge. Infants and children using a beta-blocker within 12 hours of the challenge, and short-acting, medium-acting, or long-acting anti-histamines more than once within 3, 7, or 21 days of DBPCFC, respectively, or oral steroids within 21 days of DBPCFC were excluded from the study. Adverse events were recorded throughout the study. The trial was registered in the ClinicalTrials.gov Protocol Registration System (ID number: NCT01622426).

### Sample size

In a study with a binomial outcome (reaction versus no reaction), the sample size can be determined by calculating a binomial confidence interval (CI) for p, the probability of having a reaction, as demonstrated previously [20]. In the case of 0 observed reactions, the upper 95% CI for p is less than 0.10 when the sample size is 29 participants. Thus studying at least 29 participants and having none classified as positive in the DBPCFC allows the conclusion that the study provided 95% confidence that at least 90% of children with confirmed CMA who ingest the tested formula would have no reaction [3,21]. To explore the tolerance to a new Aaf either in children with IgE or non-IgE-mediated CMA we planned to enroll at least 29 children per group.

### Study tests

#### Food allergy screening tests

Measurement of serum specific IgE against CMP (alpha-lactalbumin, ALA; beta-lactoglobulin, BLG; and casein); SPTs with whole milk, CMP, placebo formula (Neocate, Nutricia, Milan, Italy) and study formula (Sineall, Humana, Milan, Italy); and APTs with whole milk, placebo and study formula, were performed in all patients.

Serum samples from patients were analyzed for specific IgE antibody titers against CMP using a commercially available kit (CAP-RAST, Pharmacia Diagnostics AB, Uppsala Sweden). The detection limit of the system was 0.35 kU/L IgE. Subjects were deemed sensitized if their specific IgE levels exceeded the detection limit.

Skin prick tests were performed using a 1-mm single peak lancet (ALK, Copenhagen, Denmark), with histamine dihydrochloride (10 mg/ml) and isotonic saline solution (NaCl 0.9%) as positive and negative control, respectively. Reactions were recorded on the basis of the largest diameter (in millimetres) of the wheal and flare at 15 min. The SPT results were considered “positive” if the wheal was 3 mm or larger, without reaction of the negative control.

Atopy patch tests were performed as previously reported [22,23]. Briefly, we used 1 drop (50 μl) of fresh milk put on filter paper and applied with adhesive tape to the unaffected skin of the child’s back, using 12-mm aluminium cups (Finn Chambers on Scan pore; Epitest Ltd Oy, Tuusula, Finland). Isotonic saline solution was the negative control. The occlusion time was 48 h and results were read 20 min and 24 h after removal of the cups. To exclude false positive reactions, we also tested allergens in a 1:10 solution. Seventy-two hours after the start of the test, reactions were classified as follows: - negative; +/− doubtful: erythema only; + weak positive: erythema, slight infiltration, papules; ++ strong positive: erythema, infiltration, papules, little vesicles; +++ very strong positive: erythema, large confluent vesicles in bubbles. Children and their families were requested to report any delayed skin reaction that was noticed after this time. Irritant or doubtful reactions, including sharply demarcated confluent erythema, or reactions confined to margins without infiltration, were deemed negative.

#### Oral food challenge

All food challenges were performed as DBPC, and took place in the outpatients clinic on 2 separate days with a one week interval, as previously reported [24]. The results were assessed simultaneously by 3 experienced paediatric allergists in each Center. Randomization and preparation of the challenges were performed by clinical dieticians not directly involved in the study. We created a computer-generated randomization list of participant numbers indicating the order in which each study formula was used in the DBPCFC.

Briefly, every 20 minutes, successive doses (0.1, 0.3, 1, 3, 10, 30 and 100 mL) of placebo (Neocate, Nutricia, Milan, Italy) or of study formula (Sineall, Humana, Milan, Italy) were administered. Full emergency equipment and drugs (epinephrine, antihistamines, and steroids) were at hand. Study subjects were scored for 9 items divided into 4 main categories: *i.* General (lowered blood pressure plus tachycardia); *ii.* Skin (rash, urticaria/angioedema); *iii.* Gastrointestinal (nausea/repeated vomiting, crampy-like abdominal pain, diarrhea); and *iv*. Respiratory (sneezing/itching, nasal congestion/rhinorrhea, stridor deriving from upper airway obstruction or wheezing) on a 0- to 3-point scale (0, none; 1, light; 2, moderate; and 3, severe). If at least 2 of the 3 physicians independently scored any item at level 3, or 2 (or more) items at level 2, the test result was considered positive. Clinical symptoms occurring within 2 h of administering the highest dose were defined as “immediate reactions”, and those occurring more than 2 h after the highest dose were defined as “delayed reactions”. Patients were observed for 2 h after the final dose, and then discharged. In the case of a positive DBPCFC, at any testing dose, the patient remained under observation until after symptoms were resolved. If patients did not show any symptoms within the first 24 hours, to assess long-term tolerance and reveal any false-negative results to the challenges, parents were advised to administer to the patients one single top dose (100 ml) of the tested formula (*verum* or placebo) everyday at home for 7 days (7-day home feeding period). Parents were invited to record daily: the total amount of formula ingested by the subject; the presence and severity of vomiting, diarrhea, rash, runny nose, wheezing, or any other symptoms (rated as mild, moderate, or excessive); and the number of bowel movements. If any symptoms occurred during this period, the subjects returned to the outpatient clinic on the same day. The investigators completed a final evaluation at the end of the 7-day home feeding period. After 7-day home feeding period of *verum* or placebo administration, the patients were examined and the parents interviewed at the Centers. To rule out a false-negative challenge result parents were asked to contact the Center if any symptoms occurred in the following 7 days after the DBPCFC procedures. The challenge was considered negative if the patient tolerated the entire challenge, including the observation period. The composition of the study formula is provided in Table 1. 

**Table 1:** Composition of the study formula

		**100 g powder**	**100 ml (15 g powder + 90 ml water)**
**Energy**	**kJ Kcal**	**2011 480**	**302 72**
**Carbohydrates**	**g**	**58.2**	**8.7**
Dextrins	g	58.2	8.7
**Protein**	**g**	**12.2**	**1,8**
**Fat**			
Saturated fatty acid	**g**	**22.0**	**3.3**
Monounsatured fatty acid	g	7.8	1.2
Polyunsatured fatty acid	g	9.7	1.5
Linoleic acid 18:2	g	4.5	0.7
Alpha-Linolenic acid 18:3	g	3.98	0.59
Linoleic acid : Linolenic acid	g	0.5	0.076
		8	8
**Vitamins**			
Vitamin A	μg	440	66
Vitamin D	μg	9.6	1,4
Vitamin E	mg	7.5	1,1
Vitamin K	μg	23	3,5
Vitamin B_1_	mg	0.40	0,06
Vitamin B_2_	mg	0.64	0,1
Vitamin B_6_	mg	0.47	0,07
Vitamin B_12_	μg	0.97	0,15
Vitamin C	mg	63	9,5
Niacin	mg	3,8	0,57
Pantothenic acid	mg	3,8	0,57
Folic acid	μg	58	9
Biotin	μg	9,5	1,4
Choline	mg	98	15
Inositol	mg	20	3
Carnitine	mg	17.9	2,7
Taurine	mg	28	4,2
**Nucleotides**			
Cytidine-monophosphate	mg	3.8	0,6
Uridine-monophosphate	mg	4.5	0,7
Adenosine-monophosphate	mg	6.9	1,0
Guanosine-monophosphate	mg	1.3	0,2
Inosine monophosphate	mg	2.5	0,4
**Minerals**			
Sodium	mg	165	25
Potassium	mg	570	86
Chloride	mg	308	46
Calcium	mg	460	69
Phosphorus	mg	295	44
Magnesium	mg	42	6
**Trace elements**			
Iron	mg	6.9	1,0
Copper	μg	355	53
Zinc	mg	7.1	1,1
Manganese	mg	0.41	0,06
Iodine	μg	99	15
Molybdenum	μg	14.5	2,2
Fluorine	μg	0.17	0,03
Selenium	μg	9.0	1,4
Chrome	mg	14.5	2,2
**Osmolarity**	**mOsm/l**		**275**

#### Markers of intestinal mucosa inflammation

The faecal concentration of calprotectin and of ECP were determined 24 hours before and 7 and 14 days after DBPCFC to evaluate the occurrence of even subclinical intestinal mucosa inflammation in response to study formula. Stool samples were collected, stored at −20°C and analyzed at the end of the study by a researcher blinded to the results of DBPCFC. Faecal calprotectin levels were measured by a highly sensitive enzyme-linked immunosorbent assay (ELISA, Calprest®, Eurospital, Italy), and the results were expressed as μg/gr of stools. In a previous study we defined normal FC values for healthy subjects (aged >12 months) within 100 μg/gr of stools [16,25]. Eosinophilic cationic protein was analyzed by a commercially available ELISA assay (ECP ELISA kit, Dasit spa, Milan, Italy) and expressed as μg/g stools. We defined normal mean stool ECP value for healthy subjects (aged >12 months) as 0.88 μg/gr of faeces, 95% CI 0.7–1.1 (data on file).

### Statistical analysis

The SPSS software package (version 16.0 for Windows; SPSS, Inc. Chicago, Ill) was used for statistical analysis. The Pearson X^2^ test and the Fischer exact test were applied for categorical variables. Tests for equality of means were used to examine continuous variables. For all statistical tests, a 2-tailed P value of less than .05 was considered significant.

## Results

Of the 68 subjects assessed for eligibility, 60 patients (44 boys, 73.3%; median age 37, 95% CI 34.5–39.6 months) completed the study. The study population included 47 CMA subjects with multiple food allergies to hen’s egg, soy, peanut, wheat, and tree nut. The main characteristics of the study population are reported in Table 2. 

**Table 2:** Main demographic and clinical characteristics of the study population

	***IgE mediated cow’s milk allergy n=29***	***Non-IgE-mediated cow’s milk allergy n=31***
**Sex, m (%)**	20 (69)	24 (77.4)
**Age, m (95% CI)**	35 (31.0–38.9)	39 (35.7–42.3)
**Volume of milk eliciting positive challenge, ml (95% CI)**	6 (1.9–10.2)	144.4
**Time of onset of reactions, minutes (95 % CI)**	48.1 (36.1–60.0)	1843.5 (1618.3–2068.8)
**Concomitant multiple food allergies, n (%)**	24 (82.7)	23 (74.1)
·***Hen’s egg, n (%)***	16 (66.7)	5 (21.7)
·***Soy, n (%)***	3 (12.5)	11 (47.8)
·***Peanut, n (%)***	10 (41.7)	0
·***Wheat, n (%)***	5 (20.8)	8 (34.8)
·***Tree nut, n (%)***	3 (12.5)	0
**Gastrointestinal symptoms, n (%)**	12 (41.4)	16 (51.6)
·***Vomiting, n (%)***	9 (31)	11 (35.5)
·***Diarrhea, n (%)***	4 (13.8)	20 (64.5)
·***Hematochezia, n (%)***	0	2 (6.4)
**Cutaneous symptoms, n (%)**	14 (48.3)	8 (2.6)
·***Atopic dermatitis, n (%)***	10 (71.4)	8 (100)
·***Urticaria***	4 (28.6)	-
**Respiratory symptoms, n (%)**	14 (48.3)	0
**Anaphylaxis, n (%)**	6 (20.7)	0
**Positive serum specific IgE to CMP, n (%)**	26 (89.6)	0
**Positive skin prick test to CMP, n (%)**	28 (96.5)	0
**Positive atopy patch test to CMP, n (%)**	0	27 (87)

At enrolment 28 out of the 29 children with IgE-mediated CMA presented a positive SPT (28 to whole milk, 96.5%; 20 to ALA, 68.9%; 16 to BLG, 55.1%; and 8 to casein, 27.5%). All these subjects presented negative SPT to placebo or study formula. Specific IgE determinations for CMP were positive in 26 out of 29 patients with IgE-mediated CMA (89.6%). Twenty-seven out of 31 children with non-IgE-mediated CMA (87%) presented an APT positive to whole milk. One patient presented a low positivity (+) for APT to placebo. In all subjects with non-IgE-mediated CMA the APT to study formula resulted negative.

The DBPCFC and open challenge resulted negative in all subjects. In particular all patients were able to tolerate at least 100 ml of the study formula daily (mean 123 ml, range 105–143). No serious adverse events occurred during the DBPCFC, open challenge, or extended 7-day feeding period of the study Aaf. As determined by daily parental record, acceptance and tolerance of the study Aaf were good.

The optimal tolerance to the new Aaf was also suggested by the result of FC and ECP determination. All of the 60 subjects who completed the study had negative FC and ECP results even before and after the DBPCFC: FC (mean value before challenge 36.3 ± 22.1 μg/g vs mean values at 7 and 14 days after challenge 32.5 ± 23.8 μg/g and 33.5 ± 21.6 μg/g stools, respectively), and ECP (mean value before challenge 0.93 ± 0.31 μg/g vs mean values at 7 and 14 days after challenge 0.92±0.27 μg/g and 0.90±0.30 μg/g stools, respectively) remained stable after administration of the study formula.

## Discussion

This is the first study on the tolerance to this new commercially available Aaf in children with CMA. The study provided 95% confidence that more than 90% of subjects with CMA, many of whom had multiple food allergies, tolerate the new Aaf, thus demonstrating the hypoallergenicity of this formula. Skin prick tests and APTs performed with the new Aaf resulted negative in all study subjects. A peculiarity of our study is that we evaluated also children with non-IgE-mediated CMA. In this subgroup of patients tolerance was optimal. This is an important point because the majority of currently available data on Aaf has been obtained in patients with IgE-mediated CMA. Considering the substantial differences in the immunologic mechanism and clinical presentation of non-IgE-mediated CMA, we believed of importance to explore the hypoallergenicity of this formula also in these patients. Subjects with non-IgE-mediated CMA frequently present with gastrointestinal symptoms [26]. All patients with these characteristics tolerated the new Aaf. Of particular importance in patients with gastrointestinal symptoms related to non-IgE-mediated CMA could be the lower osmolarity, compared to other Aafs on the market; and the presence of nucleotides concentration similar to that observed in human milk [27]. Extensive evidence indicates that dietary sources of nucleotides are important to support immune function, small intestinal development and function, and other processes of rapid cell growth [27-29]. However, future clinical studies examining the effects of dietary nucleotides as single agents are necessary to better understand the role that nucleotides play in children with food allergy-related enteropathy as well as to demonstrate the expected clinical benefits.

According to the optimal clinical tolerance to this new Aaf we demonstrated that faecal levels of calprotectin and ECP remained stable within the normal range during the challenge.

As other studies conducted on Aafs we need further studies to investigate the long term effects of this dietary treatment on body growth and development, and on the time of tolerance acquisition in children with CMA.

## Conclusions

We conclude that this new Aaf is well tolerated by children with IgE- or non-IgE mediated CMA, and it could be used as a safe dietotherapy regimen for children with this condition.

## Competing interests

This trial was funded in part through a grant from Humana, Milan Italy devolved to the Department of Pediatrics of the University of Naples “Federico II”. Humana staff did not participate in the protocol development, study oversight, regulatory reporting, monitoring the progress of the study, or manuscript preparation. In addition, Humana staff did not have access to outcome data until the trial was closed. The authors alone are responsible for the content and writing of the paper.

In the previous 5 years the research group received funding from: Italian Ministry of Health, Italian Ministry of University and Scientific Research, Italian Agency of Drug (AIFA), National Foundation of Thermals, Mead Johnson Nutritionals, Campania Region. No other potential conflicts of interested were reported.

## Authors’ contributions

RBC, RN and RT conceived the study, and participated in its design and coordination and wrote the manuscript. LL, LC, MDC, AP, AC cared the patients. GT participated in the design of the study and performed the statistical analysis. All authors read and approved the final manuscript.

## Pre-publication history

The pre-publication history for this paper can be accessed here:

http://www.biomedcentral.com/1471-2431/13/24/prepub
